# Tissue Engineering Meets Nanotechnology: Molecular Mechanism Modulations in Cornea Regeneration

**DOI:** 10.3390/mi12111336

**Published:** 2021-10-30

**Authors:** Olja Mijanović, Timofey Pylaev, Angelina Nikitkina, Margarita Artyukhova, Ana Branković, Maria Peshkova, Polina Bikmulina, Boris Turk, Sergey Bolevich, Sergei Avetisov, Peter Timashev

**Affiliations:** 1 Institute for Regenerative Medicine, Sechenov University, 8-2 Trubetskaya St., 119991 Moscow, Russia; nikitkinaangelina@gmail.com (A.N.); margarita.artyukhova@gmail.com (M.A.); peshkova_m_a@staff.sechenov.ru (M.P.); bikmulina_p_yu@staff.sechenov.ru (P.B.); boris.turk@ijs.si (B.T.); timashev_p_s@staff.sechenov.ru (P.T.); 2 Saratov Medical State University N.A. V.I. Razumovsky, 112 Bolshaya Kazachya St., 410012 Saratov, Russia; pylaev_t@ibppm.ru; 3 Institute of Biochemistry and Physiology of Plants and Microorganisms, Russian Academy of Sciences, 13 Prospekt Entuziastov, 410049 Saratov, Russia; 4 Department of Forensic Engineering, University of Criminal Investigation and Police Studies, 196 Cara Dušana St., Belgrade 11000, Serbia; ana.brankovic@kpu.edu.rs; 5 World-Class Research Center “Digital biodesign and personalized healthcare”, Sechenov University, 8-2 Trubetskaya St., 119991 Moscow, Russia; 6 Department of Biochemistry and Molecular and Structural Biology, Jožef Stefan Institute, 1000 Ljubljana, Slovenia; 7 Faculty of Chemistry and Chemical Technology, University of Ljubljana, 1000 Ljubljana, Slovenia; 8 Department of Human Pathology, Sechenov University, 8-2 Trubetskaya St., 119991 Moscow, Russia; bolevich2011@yandex.ru; 9 Department of Eye Diseases, Sechenov University, 8-2 Trubetskaya St., 119991 Moscow, Russia; s.avetisov@niigb.ru; 10 Research Institute of Eye Diseases, 11 Rossolimo St., 119021 Moscow, Russia; 11 Chemistry Department, Lomonosov Moscow State University, Leninskiye Gory 1-3, 119991 Moscow, Russia

**Keywords:** cornea regeneration, tissue engineering, nanotechnology, molecular mechanisms

## Abstract

Nowadays, tissue engineering is one of the most promising approaches for the regeneration of various tissues and organs, including the cornea. However, the inability of biomaterial scaffolds to successfully integrate into the environment of surrounding tissues is one of the main challenges that sufficiently limits the restoration of damaged corneal tissues. Thus, the modulation of molecular and cellular mechanisms is important and necessary for successful graft integration and long-term survival. The dynamics of molecular interactions affecting the site of injury will determine the corneal transplantation efficacy and the post-surgery clinical outcome. The interactions between biomaterial surfaces, cells and their microenvironment can regulate cell behavior and alter their physiology and signaling pathways. Nanotechnology is an advantageous tool for the current understanding, coordination, and directed regulation of molecular cell–transplant interactions on behalf of the healing of corneal wounds. Therefore, the use of various nanotechnological strategies will provide new solutions to the problem of corneal allograft rejection, by modulating and regulating host–graft interaction dynamics towards proper integration and long-term functionality of the transplant.

## 1. Introduction

The human cornea is a complex five-layer structure that both protects the eye and refracts light, contributing greatly to the eye’s optical power. Proper light refraction is ensured through collagen fibrils’ special organization in the three inner layers of the cornea [1]. For example, in Bowman’s membrane collagen fibrils are randomly organized and tightly woven; at the same time, they form multiple lamellae in the stroma and hexagonal lattice in Descemet’s membrane [2]. This unique microarchitecture, on the one hand, maintains corneal shape, and on the other hand ensures transparency, these two factors being essential for proper light refraction [1]. 

Various mechanical, chemical or thermal traumatic factors can impair cornea integrity and homogeneity [1]. The corneal epithelium, the outermost layer of the cornea, undergoes constant self-renewal due to the proliferation and migration of populations of the progenitor limbus cells located at the cornea and sclera border [3], and has good regenerative properties, allowing self-healing of superficial corneal injuries. However, deeper corneal damage can lead to severe vision impairment, requiring a corneal transplant.

Currently, the only approved treatment strategy for corneal damage is keratoplasty [2,3]. However, the lack of donor tissue, transplant rejection, and various complications following the surgery significantly reduce the effectiveness of the procedure [4,5]. In recent years, in order to solve the problem of cornea allograft deficiency, attention had been drawn to artificial cornea manufacturing using various tissue engineering approaches [6]. 

Despite significant advances, the engineering of corneal tissue still faces many limitations and has a number of disadvantages [7]. One of the main limitations of artificial corneal transplants is poor integration with native host tissues and high rejection rates due to various immune responses in native corneal tissues, which are especially acute in the damaged area [8]. The problems of interactions between a tissue-engineered (TE) graft and the host microenvironment will be further discussed in this review, as well as the recent studies on the multiple molecular mechanisms modulating the processes of healing, inflammation, and remodeling of the extracellular matrix (ECM) [9] and cell death [10,11,12].

State-of-the-art nanotechnology-based methods seem to be able to help in modulating these molecular mechanisms that are crucial for successful graft integration, thus helping to overcome some existing drawbacks in corneal tissue engineering. Current research on corneal regeneration by the means of nanotechnology and nanomedicine implies the use of nanocarriers for drug delivery, gene therapy agents [13], etc., as well as the use of nanostructured matrices to improve cell adhesion and proliferation. Many studies were devoted to the development of effective strategies for intracorneal nanomaterial (NM)-based delivery of various biomolecules, such as DNA [14], antibodies [15], peptides [16], and therapeutic agents [17]. In addition, extensive research has been conducted in the field of “smart” biocompatible nanoscaffolds, implying synthetic and semi-synthetic biomaterials [18] with desired properties and customizable structures designed for specific tasks.

Modulating molecular interactions between native cells and the transplanted biomaterial through various physicochemical [19] and nanotechnological [20] approaches will help to guide the healing processes in the damaged corneal tissues and improve clinical outcomes in patients with corneal injuries and diseases. 

## 2. Corneal Tissue Engineering

With the background of restricted possibilities of traditional methods for cornea regeneration, transplantation, and hardly accessible donor cornea, novel methods are in high demand. The implementation of biomaterials opens a new field in approaches to cornea regeneration. 

### 2.1. Different Approaches for Cornea Replacement

The widespread technique of organ replacement with xenogenic, decellularized, ECM-enriched matrices allows for the preservation of the native composition and anatomy of the target tissue. Such matrices usually do not contain any cells, but support the regeneration of host cells. The mechanical and optical properties of the decellularized cornea (DC) are similar to the native one [21,22]. Another example of a decellularized matrix for cornea regeneration is an amniotic membrane (AM). An AM has a three-layered structure that resembles the corneal epithelium structure and has great regenerative and anti-inflammatory properties [23]. However, both DC and AM have strong disadvantages as a cornea analog. These include the possibility of a strong immune response to remaining collagen fibrils, the possible transmission of infectious diseases, changes in stroma structure, transparency and biomechanical stability during the material preparation, and lack of complete re-cellularization after implantation [23,24,25,26,27]. 

The polar opposite approach implies the creation of a native-resembling environment de novo. Unique properties of either synthetic or natural biomaterials often provide tunability required for cornea regeneration. For instance, fibrin glue is used to restore corneal integrity after frequent intraoperative and postoperative corneal traumas and perforations [28,29]. A chemically modified UV crosslinkable material based on GelCORE gelatin has been developed which mimics the natural stiffness of the cornea and is highly adhesive, cytocompatible, and biodegradable. The hydrogel was able to seal corneal defects without the need for suturing and promoted re-epithelialization of the corneal surface [30]. Clinically available synthetic corneas are widely used to replace donor ones, including keratoprosthesis (KPro made from poly (methyl methacrylate) (PMMA) [31] and AlphaCor^TM^ poly (2-hydroxyethyl methacrylate) (PHEMA) [32]. However, natural biomaterials such as fibrin and gelatin often face rapid degradation rates, weak mechanical properties, and can act as a physical barrier for the migrating epithelial cells [33,34]. Serious side effects of artificial corneas based on synthetic matrices were reported, among them an acute foreign body response and hyperacidity of degradation products, which lead to corneal scarring [35]. 

To overcome existing obstacles, numerous approaches for the modification of biomaterials can be applied. ECM-containing matrices can be “strengthened” by crosslinking [36] or by combining with other materials, such as PCL nanofibers [37]. To improve biocompatibility, synthetic materials are also combined with natural biopolymers, e.g., PCL combined with collagen, gelatin, or chitosan [35,38,39,40]. Therefore, the combination of various biomaterials is the most promising strategy for cornea regeneration in terms of biocompatibility, mechanical properties, transparency, immune response, and cell behavior. 

The tissue engineering (TE) approach satisfies these inquiries perfectly since it combines biomaterials, biochemical factors, and cells to form tissue-like structures. Corneal TE has attracted great interest recently due to avoiding many of the complications encountered in traditional donor corneal transplantation. The involvement of cells allows not only the creation of a cornea analog, but also give promises for the full regeneration and integration of the graft. 

### 2.2. Cornea TE Grafts

#### 2.2.1. Hydrogel-Based Grafts 

Since collagen is the major protein in the cornea ECM, it is widely used both as a base and in addition to hydrogel grafts for cornea regeneration. Collagen vitrigel is widely used for the construction of corneal equivalents [41] and the treatment of corneal endothelial dysfunction [42]. Wang et al. cultured primary human corneal endothelial cells (HCECs), exhibited elongated morphology, and increased expression of corneal endothelial markers ZO-1 and Na^+^/K^+^ -ATPase on a collagen vitrigel [35]. Crosslinking collagen, e.g., with riboflavin (RF), is widely used to significantly improve its mechanical stiffness and chemical stability [19,43,44,45]. Fang Chen et al. [46] stitched collagen and HA together directly in a rabbit cornea wound in situ without a catalyst or light activation. The growth of corneal epithelial cells on the gel surface was maintained for 7 days, and no inflammation was found in the surrounding tissue [46].

Gelatine-based materials often have good transparency due to their high water content, which makes them a promising candidate for use in ocular tissue engineering [47]. Goodarzi et al. [48] studied the possibility of using a hydrogel based on type I collagen and crosslinked EDC/NHS gelatin, as an equivalent of the cornea, in which the MSCs of human bone marrow were encapsulated. The results show that the inclusion of COL-I increases optical properties, hydrophilicity, rigidity, and Young’s modulus. 

An alginate-chitosan hydrogel was created for the transplantation of LSC cells for corneal reconstruction after alkaline corneal burns. LSC cells cultured in vitro expressed stemness marker p63, but did not show expression of differentiating epithelial markers of cytokeratin 3 and 12. However, a significant improvement in epithelial repair was shown [49]. To enhance the mechanical properties of alginate, PCL matrices and PCL/chitosan electrospinning matrices were incorporated into alginate hydrogels for the treatment of corneal lesions [50].

Thermosensitive hydrogels based on chitosan were demonstrated as a promising treatment strategy for alkaline corneal burns. This approach reduced the inflammatory and apoptotic processes in the damaged corneal tissues [51]. The inclusion of stromal cell factor-1 alpha (SDF-1 alpha) in a thermosensitive chitosan-gelatin hydrogel improved the regeneration of the epithelium of the corneal damaged by alkali; LESCs expressed the characteristic marker ∆Np63. The formation of a dense epithelium occurred due to stem cell homing and the secretion of growth factors through the axis of chemokines SDF-1/CXCR4 [52].

Hyaluronic acid (HA) can be a favorable addition to hydrogel composition due to its viscosity, biocompatibility, biodegradability, non-toxicity, and significant mucoadhesive properties [53]. Moreover, HA suppresses the expression of inflammatory cytokines and increases the expression of anti-inflammatory cytokines associated with tissue repair and healing [54]. The negative charge of HA promotes adhesion on the ocular surface, contributing to a longer therapeutic effect and allowing drug molecules to permeate the cells of the mucous epithelium [53].

#### 2.2.2. Membrane- and Film-Based Grafts 

Another strategy for cornea TE is utilizing flat surfaces for reducing the graft thickness, which is crucial for the specific structure of the cornea. The fibroin membrane is able to support the formation of a multi-layered epithelium and the growth of human corneal limbal epithelial (hCLE) cells, and is currently considered a standard substrate used for corneal epithelial cell transplantation [55]. When hybrid films based on tropoelastin were constructed, the obtained membranes were optically transparent, permeable to glucose, and also supported the growth and function of epithelial and endothelial cells [56]. To increase the transparency of the corneal equivalent, hydroxypropyl methylcellulose (HPMC) was introduced into collagen to create transparent matrices with a high rate of light transmission [45]. The permeability for glucose, tryptophan, and NaCl was high in such membranes and similar to the native human cornea. This membrane supported the adhesion and proliferation of human corneal epithelial cells (HCECs). Seven months after the implantation of collagen-HPMC membranes into the cornea of rabbits, high optical transparency and growth of stromal keratocytes were maintained [45]. Wenhua Xu et al. [57] developed a membrane based on carboxymethylchitosan, hyaluronic acid, and gelatin as a carrier for primary rabbit corneal epithelial cells (CEpCs). A cell construct has been used to treat alkali-induced corneal damage in rabbits. The resulting membrane was found to be transparent, biodegradable, and suitable for CEpC attachment and proliferation. As noted above AM also showed potential as a mechanical support of transplanted limbal stem cells (LSCs) [37], limbal epithelial stem cells (LESCs) [58], corneal endothelial cells (CEnCs) [59], CSC cells [60], and CEC cells [61].

### 2.3. Modulation of Cell Behavior by Cornea TE Grafts

Newer studies have shown that neighboring mechanical surroundings (the structure, composition, and compliance of the extracellular matrix) strongly influence the behavior of LESCs. Recently, it was shown that the efficiency of cell differentiation probably depends on the biomaterials used and the composition of the cultured medium. The closer the environment resembles the human cornea, the higher the likelihood that most of the MSCs differentiate into corneal keratocytes [62]. Therefore, some authors have proposed the modulation of tissue biomechanics (e.g., substrate stiffness) as a pharmacological method for modulating the phenotype of cells, initiating novel prospects for establishing more successful cell therapies and medical devices for cornea tissue regeneration [63]. For instance, fiber orientation and composition have a significant impact on the behavior of cells in the scaffold [64]. Julia Fernández-Pérez et al. [64] obtained matrices based on decellularized corneal ECM and PCL by electrospinning to mimic the fibrous structure of the cornea. Fiber alignment and ECM incorporation influenced cell morphology and migration but did not significantly affect the phenotype. Keratocyte markers were increased in all types of scaffolds compared to TCPS. 

Therefore, the TE approach provides plenty of cues vital for the establishment of the full-fledged cornea analog. Among them are high optical transparency, mechanical integrity, and proper cell behavior, leading to effective re-epithelialization. However, biocompatibility with host tissues remains crucial for preventing infection and promoting implant integration [65]. 

## 3. Molecular Pathways and Interactions between Host Tissues and a Graft

In response to biomaterial implantation, cascades of molecular pathways are triggered. They determine the success of graft integration as well as its biological activity and functionality. Degradation products released by TE scaffolds, as well as subsequent changes in the characteristics of the biomaterial surface, activate an immune response in the host tissues [66]. Biomaterials frequently induce adverse immune responses in host tissues, which result in extensive inflammatory reactions, impaired healing processes, fibrous encapsulation, and rejection of the TE construct [67]. The main strategies for healing and launching regenerative processes in corneal cells have focused on such areas as modulation of the immune response, prevention of angiogenesis, and the modulation of cell interactions [2]. In order to develop efficient strategies to overcome the undesirable biological interactions with transplanted biomaterial, a deeper understanding of the interplay between the transplant and the native host environment, as well as the damage and graft-induced alterations in molecular signaling, is required. 

### 3.1. Processes Involved in Cornea Healing

#### 3.1.1. ECM Reorganization and Re-Epithelization

The process of corneal wound healing is regulated by the interplay between the corneal epithelium, the Bowman layer, and the corneal stroma. An important role in this process is played by ECM and dissolvable factors produced by corneal epithelial cells and keratocytes [68]. Damage to epithelial cells may result in pathological ECM reorganization. Keratocytes around the site of injury trigger apoptosis and many of them transdifferentiate into fibroblasts. Some fibroblasts will produce α-SMA and become myofibroblasts under the influence of TGF-β and other soluble factors [69]. These non-transparent cells produce large amounts of disorganized ECM in the anterior part of the stroma, eventually hazing it and leading to a loss of corneal transparency [70]. Dysfunction in a group of signaling transduction pathways, e.g., Wnt signaling pathway (or JAK/STAT, MAPK, and PI3K/Akt signaling pathways), triggers the pathological transdifferentiation of a corneal epithelium into a skin-like epithelium [71], which results in impaired corneal regeneration. Guo et al. discovered that miR-10b (the Wnt signaling pathway) and three intersection genes (dedicator of cytokinesis 9, neuronal differentiation 1, and activated leukocyte cell adhesion molecule) may cooperate and play a key role in the process of transdifferentiation. The changes in ECM organization are perceived by transmembrane surface proteins, such as integrins, that result in the activation of various intracellular signaling cascades, mainly the focal adhesion kinase (FAK)–Src complex [72]. Activation of the FAK–Src pathway leads to re-epithelialization of the injured tissue. A sharp increase in the expression of matrix metalloproteinases (MMPs) and proteases is observed in the process of corneal wound healing. MMPs are also associated with the degradation of type I, II, and III collagen, a major ECM component. The expression of MMPs in the cornea is modulated by cytokines (such as IL-1b and IL-6) and growth factors (such as TGF-β) through tuning the expression of several transcription factors, such as AP-1 and Sp1 [73,74]. 

#### 3.1.2. Soluble Factors

Growth factors (GF) play a pivotal role in corneal regeneration. Platelet-derived GF (PDGF), transforming GF beta (TGF-β), and hepatocyte GF (HGF) were shown to play a key role in modulating cell proliferation and myofibroblast differentiation [2]. Studies have indicated that HGF promotes the proliferation of CECs. Moreover, HGF treatment reversed the antiproliferative effect of IL-1β in vitro, indicating that HGF actively suppressed the inflammatory environment in the corneal epithelium. On the other hand, HGF significantly reduced the infiltration of DC45+ inflammatory cells in the cornea [2,75]. Salabarria et al. showed that local VEGFR1/R2 trap treatment prior to transplantation increases transplantation success. This treatment suppresses corneal tissue infiltration with CD11c+ dendritic cells and stimulates the local expression of pro-inflammatory and immune-regulatory cytokines [76]. 

#### 3.1.3. Oxidative Stress

Endothelial cell loss after corneal transplantation may be caused by oxidative stress and endoplasmic reticulum (ER) stress [12]. The mechanism of oxidative-stress-induced apoptosis starts when inflammatory cytokines promote the production of reactive oxygen species which set off permeabilization of the mitochondrial membrane as well as the NF-κB signaling pathway [12]. NF-κB signaling pathway activation stimulates the aging of vascular endothelial cells. The ER stress mechanism is also triggered by cytokines. It causes apoptosis through the TGF-β signaling pathway [12].

### 3.2. Modulation of Cornea Regeneration by Biomaterials

#### 3.2.1. Re-Epithelization

Recent studies show that mechanical properties, including rigidity, stiffness, and elasticity, affect cell behavior, as well as their ability to adhere, proliferate, and differentiate [64]. The orientation of biomaterial fibers and their composition also have a significant impact on the biocompatibility, inflammation, neovascularization, and cell behavior on the scaffold [77,78]. 

Rapid re-epithelialization is critical to prevent infection and promote implant/host integration. Previously described in vitro studies have shown that biomaterial mechanics and surface roughness affect the migration and maturation of epithelial cells [63]. To address the problem of re-epithelialization, Wang et al. added a thin, structurally uniform biosynthetic Bowman membrane of non-lamellar amorphous collagen I over the collagen layer of the corneal stroma to create a bilayer equivalent of the cornea. Epithelial cells formed multilayer structures on top of sBM and expressed key markers of limbal stem cells and epithelial cells p63, K3, K12, K14, and tight junction protein ZO-1 [12].

#### 3.2.2. ECM Analogs

Corneal cells actively interact with implanted biomaterials. For instance, ECM adhesion proteins, such as fibronectin and vitronectin, adhere to the surface of the biomaterial and play an important role in modulating the inflammatory response to the biomaterial [79]. Fibronectin and vintronectin enhance cell adhesion [80,81], promote macrophage fusion, and participate in the chronic phase of a foreign body response (FBR) [82,83]. 

#### 3.2.3. Mechanical Properties

The stiffness of the TE corneal constructs affects cellular spatial migration and the phenotype. This downstream signaling includes RhoA and Rho-kinase proteins that modulate the cytoskeletal structure by inducing contractility or migration through actin and myosin [84]. Some authors suggested that the modulation of tissue biomechanics may present a controlling mechanism for pharmacological control of CEC phenotypes [63]. Gouveia et al. demonstrated that soft substrates, similar to the limbus, stimulate cell proliferation and stratification without influencing cell survival. They proposed that soft substrates induce YAP inactivation and keep ΔNp63, β-catenin, and ABCG2 expression levels high. ΔNp63 inhibits YAP and Wnt/β-catenin signaling and, at the same time, activates Sox9, which enhances the expression of stem cell markers such as ABCG2 and CK15. Next, β-catenin promotes pro-proliferation factors (e.g., Ki67, cyclin D1, and Myc) and inhibits BMP4 expression. As stratification progresses, the role of soft substrates decreases and YAP activates, leading to cell differentiation [85].

#### 3.2.4. Surface Properties and Topography

Interaction of the biomaterial surface with adsorbed proteins is crucial in the immune response to the implant [86]. Various methods of altering surface chemistry have been tested to create poorly adhesive surfaces in order to control the amount, composition, and conformational changes of bounded proteins [87]. The immune system has developed the ability to recognize hydrophobic components in biomolecules as a universal molecular pattern associated with damage, thereby triggering pattern recognition receptors and leading to biological elimination [88]. The average unfolding of a protein molecule [89] and total spreading [90] are greater on hydrophobic than on hydrophilic surfaces, where proteins retain their inherent secondary structure and show little or no adsorption on the biomaterial surface [91]. To neutralize the immunogenic effects of hydrophobic surfaces, scaffolds can be modified with hydrophilic molecules such as poly(ethylene oxide) (PEO) and PEG [79]. Additionally, the surface chemistry of a biomaterial can be changed by attaching hydrophilic functional groups such as -COOH, -OH, or -NH2, allowing the regulation of protein adsorption, complement activation, and immune cell adhesion on the surface of the material [92]. Recently, researchers succeeded in the preservation of the native 3D conformation (since unfolding or misfolding of the protein molecule itself can cause adverse reactions) instead of excluding any interaction of the graft with the surrounding tissue [93].

A surface charge is another important modulator of the host immune response. Positively charged particles promote extensive activation of the inflammatory cascades, while negatively charged surfaces tend to activate a strongly pro-inflammatory innate immune response [79,94]. Particles with a negatively charged surface can inhibit the severity of the immune response by preventing antigen-presenting cells (APCs) from processing and presenting an antigen (biomaterial) for recognition by T cells [95].

Biomaterial surface topology provides a powerful tool to control and regulate corneal cell behavior [96], including cell adhesion [97], density, spreading, mobility [98], proliferation, differentiation [99], cytokine and ECM secretion [100,101], and cell signal transduction [102]. Importantly, the differentiation of keratocytes into myofibroblasts is triggered by the surface topography [103]. Thus, the surface topology of the biomaterial can inhibit the TGF-β-induced differentiation of myofibroblasts and prevent the development of fibrosis and corneal opacity during the healing process. Moreover, the differentiation of keratocytes into myofibroblasts is regulated by surface topography. Myrna et al. found that transformation into myofibroblasts could be prevented by cultured keratocytes on patterned grooves with a 1400-nm-wide pitch [103].

#### 3.2.5. Anti-Oxidative Properties

Since extensive oxidative stress can occur in the implantation site, antioxidant properties of the biomaterial would be helpful. High-molecular-weight HA [104] and chitosan [105] have intrinsic anti-inflammatory properties due to their ROS-scavenging abilities. 

#### 3.2.6. Immune Cells

Activated neutrophils are recruited from the peripheral bloodstream by chemoattractant factors, adhere at the implantation site (via β2 integrins), and try to degrade the biomaterial by phagocytosis, proteolytic enzymes, and reactive oxygen species [79]. Increased immunomodulatory cytokines IL-10 and IL-17 are critical for corneal graft survival [74]. Treatment with T regulatory cells (Tregs) or tolerogenic APCs induced by immunoregulatory factors can help restore immune privilege and thus lead to the long-term survival of the corneal allograft in high-risk recipients. Host alloimmunity is the main cause of loss of donor CEnCs after corneal transplantation [106]. Tregs play a critical role in suppressing immune responses after tissue transplantation. Tregs from low-risk hosts can protect CEnCs from both Teff-mediated and IFN-γ- and TNF-α-induced cell death. This function is significantly compromised in Tregs derived from high-risk hosts. The cytoprotective role of Tregs is mediated by the immunomodulatory cytokine IL-10; hence, IL-10 is effective in protecting CEnC from inflammatory cytokines during cell death [106]. 

Keeping in mind all the data discussed above, it can be concluded that the number of molecular signaling pathways activated in the response to corneal trauma and biomaterial implantation play a pivotal role in the immunological response to the transplant. Modulating the activation/inhibition of involved molecular pathways along with proper biomaterial composition, surface topology and other parameters may provide a solution for establishing optimal host–graft interaction and ensure successful tissue regeneration, graft integration, and long-term survival. 

## 4. Nanotechnology in Corneal Tissue Engineering 

Nanotechnologies can be used at the stage of corneal scaffold fabrication to improve their physicochemical properties, but also after scaffold implantation, for example to deliver various therapeutic agents by means of nanocarriers in order to solve the problems of inflammation, secondary infections, and neovascularization in the damaged area. 

### 4.1. Nanostructured Matrices

Nanoscaffolds possess unique mechanical properties that facilitate gas and nutrient exchange as well as the removal of cellular waste, and which also promote cell adhesion, proliferation, and differentiation [107]. For example, nanostructured ~10 nm dendrimers are high-contrast polymers that have a 3D ionic shape with numerous end groups. The biggest advantages of dendritic systems are the high density of functional side chains, the ability to manage network crosslinks, and scalability over a wide size range [108]. Dendrimer-based hydrogels have been shown to promote the rapid healing of corneal wounds without scarring or inflammation [109]. Due to the ability to control the crosslinking process and change the crosslinking chemistry, it is possible to manipulate the period of resorption, and thus control the process of wound healing on a bigger time scale. Thus, dendrimers are labile “smart” NMs and can be used for wound healing during long recovery periods with a low chance of an inflammatory response [110].

Another promising direction is the combined application of nanotechnology and corneal tissue engineering with natural biomaterials. For example, to form a biomaterial with the desired properties it can be combined with metal nanoparticles, graphene oxide, carbon nanotubes, and nanoliposomes [111]. Nanostructured hydrogels are mainly used for the delivery of genes and proteins. In situ transition from sol to gel promotes their role in enhancing the growth and functionality of other stem cells [112]. Soft nanoparticles can interact with polymer chains and can contribute to further crosslinking of the hydrogel grid to improve its mechanical properties [113]. 

### 4.2. Nanocarriers for Intracorneal Drug Delivery 

Nanocarriers can improve the bioavailability and bio-distribution of therapeutic molecules, at the same time promoting targeted delivery and controlled drug release [114]. 

The problem of secondary infections after scaffold implantation can be addressed via the application of antibiotics, anti-viral, or anti-fungal drugs encapsulated within nanocarriers. For example, speaking about anti-viral drugs, dipeptide-acyclovir-based prodrugs encapsulated in poly (lactic-glycolic acid) (PLGA) nanoparticles showed increased efficacy due to improved drug release kinetics [115], while liposomes loaded with idoxuridine were reported to demonstrate increased penetration of the drug into the cornea [116]. There is also evidence that the retention time of the antifungal drug natamika delivered via chitosan nanoparticles in the corneal epithelial layer is 1.5 times longer than when using a commercial treatment method [117]. Speaking about antibiotics, quinolones moxifloxacin [118,119], sparfloxacin [120], and levofloxacin [121] demonstrated increased bioavailability when delivered via nanocarriers; better corneal permeability was reported as well. Sometimes nanoparticles can even be used as an alternative to antibiotics, for example, as silver ones can, known for their remarkable antiseptic properties [122].

Another important issue in corneal tissue engineering is inflammation and subsequent neovascularization, which threatens corneal transparency. Currently available options to avoid this condition include corticosteroids, non-steroidal anti-inflammatory eye drops, photodynamic therapy, photocoagulation, and antibodies (bevacizumab) against vascular endothelial growth factor A (VEGF-A). These methods aim to suppress angiogenesis by blocking angiogenic factors such as VEGF, PDGF, major fibroblast growth factor (FGF), MMPs, and interleukins [123,124,125]. For example, in a study conducted by Iriyama et al., micelles consisting of a copolymer and plasmid DNA expressing the soluble VEGF receptor 1 (sFit-1) were used for gene therapy [126]. SFit-1 expression acted as a VEGF receiver and prevented activation of the angiogenesis cascade. The results showed that the injection of micelles containing a reporter gene lead to delayed sFit-1 expression and inhibition of corneal neovascularization. Gold nanoparticles were also reported to inhibit angiogenesis [127] and corneal neovascularization [128] by suppressing the vascular expression of endothelial growth factor (EGF) receptor-2.

Dexamethasone, a widely used anti-inflammatory steroid drug, was reported to show higher bioavailability and better corneal penetration when delivered via nanomicelles [129] or encapsulated within nanoparticles [118,130], while hydrocortisone, another widely used steroid drug, was reported to demonstrate fewer dose-dependent side effects when administered in the form of nanosuspension [131]. 

Rapid re-epithelialization is one of the key factors preventing infection and promoting implant/host integration. Xuan Zhao et al. used the complexes of AuNPs and microRNA-133b sorbed on collagen matrices to restore the cornea and inhibit scarring [132], reporting good and fast re-epithelialization.

## 5. Outlook and Future Perspectives 

**Figure 1:**
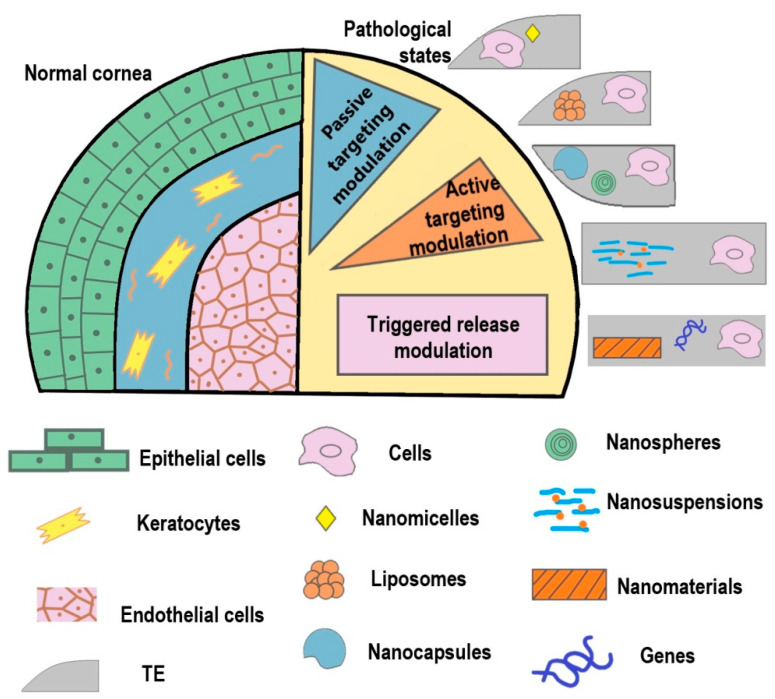
Nanomaterial-based approach in the combined therapy. Top left – healthy corneal tissue. The cornea is a complex vascular-free structure composed of five layers, three of which are of cellular nature (epithelium, stroma and endothelium). Throughout the lifetime, corneal cells are exposed to various traumatic and damaging factors from the external environment and inner disturbances in organism’s functionality. These pathological processes and damaging agents can compromise the integrity of the cornea and lead to vision loss. Top right – pathological corneal tissue. Due to the lack of modern approaches that allow the full restoration of the cornea tissue and vision, new treatment and therapeutic strategies are needed to be introduced. TE- and nanotechnology-based strategies can become a new chapter in the cornea restoration. TE constructs can act through active and passive targeting and controlled triggered release promoting the most effective approach for each set of specific molecular mechanisms and cellular events.

Regenerative ophthalmology is a rapidly evolving new field for the regeneration of lost or damaged eye cells and tissues as well as the treatment of vision loss and blindness caused by various ocular diseases, injuries, or infections. However, the cell therapy approaches in regenerative medicine are still at an early stage of development and face numerous serious problems and challenges. Effective methods and biomaterials for transplantation should support the correct rate of cell adhesion, proliferation, and differentiation, and sustain the desired cellular phenotype, cell-specific signaling, and biochemical properties. The use of combination therapy of nanomedicine/bioengineering in ocular regeneration is promising in overcoming these difficulties [133]. The possibility to introduce controlled and customizable changes in the psychochemical characteristics, size, and surface chemistry of NMs allows the construction of various matrixes with desired properties, perfectly optimized for specific biological applications (Figure 1). Non-viral gene nanocarriers such as polyplexes, mesoporous NPs, organic–inorganic hybrid nanocarriers, nanoscripts, self-organizing DNA nanostructures, and magnetic NPs are becoming promising tools for reprogramming cells on the way to treat and regenerate damaged corneal tissues. The combination of nanotechnology and immunoengineering to modulate the innate and adaptive immune response will be crucial for corneal wound healing. 

Personalized medicine that adapts the treatment of a disease based on an individual’s genetics and specific pathophysiological processes taking place in their specific case to achieve a better clinical outcome is an emerging and growing field in ophthalmology. The use of nanotechnology can allow personalized therapy and optimized drug dosages for more accurate and effective treatment of corneal diseases. It is expected that in the future nanotechnology will be used to personalize regenerative medicine using human stem cells and provide therapeutic tools to maintain a healthy environment for the growth and maturation of stem cells in the damaged area [134]. However, research in nanotechnology for regenerative ophthalmology is still at an early stage, and there is a very limited number of in vivo studies. The behavior of corneal cells on TE constructs in the area of corneal damage has been widely demonstrated in vitro, but many open questions remain due to the lack of in vivo proof-of-concept studies.

## Abbreviations

3D	Three-dimensional
ABCG2	ATP-binding cassette super-family G member 2
AM	Amniotic membrane
BMP4	Bone morphogenetic protein 4
CECs	Corneal epithelial cells
CenCs	Corneal endothelial cells
DC	Decellularized cornea
ECM	Extracellular matrix
ER	Endoplasmic reticulum
FAK	Focal adhesion kinase
hCECs	Human CECs
LESCs	Limbal epithelial stem cells
LSCs	Limbal stem cells
MMP	Matrix metallopeptidases
MSCs	Mesenchymal stem cells
NM	Nanomaterials
NPs	Nanoparticles
PCL	Polycaprolactone
PEG	Polyethylene glycol
PMMA	Poly (methyl methacrylate)
PLGA	Poly lacto-glycolic acid
RF	Riboflavin
RhoA	Ras homolog family member A
sFit-1	Soluble VEGF receptor 1
TE	Tissue-engineered
Tregs	T regulatory cells
VEGF-A	Vascular endothelial growth factor A
YAP	Yes-associated protein
α-SMA	Smooth muscle alpha-actin (alpha smooth muscle actin)
